# Detection of *Leishmania donovani* DNA within Field-Caught Phlebotomine Sand Flies (Diptera: Psychodidae) in Three Cutaneous Leishmaniasis Endemic Foci of Kurunegala District, Sri Lanka

**DOI:** 10.1155/2021/6650388

**Published:** 2021-04-09

**Authors:** Tharaka Wijerathna, Nayana Gunathilaka, Kithsiri Gunawardena, Yoshito Fujii, Deepa Gunasekara

**Affiliations:** ^1^Department of Parasitology, Faculty of Medicine, University of Kelaniya, Ragama, Sri Lanka; ^2^Visiting Scholar, Department of Parasitology, Faculty of Medicine, University of Kelaniya, Ragama, Sri Lanka; ^3^Department of Biochemistry and Clinical Chemistry, Faculty of Medicine, University of Kelaniya, Ragama, Sri Lanka

## Abstract

Leishmaniasis is a parasitic infection transmitted through the bite of female phlebotomine sand flies. Microscopy is the gold standard to detect parasites within the sand flies and for vector incrimination. However, molecular-based detection has become more popular nowadays in the identification of *Leishmania* parasites since it provides detection and species identification simultaneously with no need of laborious procedures. The entomological surveys were conducted monthly from May to October 2017 using standard entomological techniques. Field-caught sand flies were identified to the species level followed by DNA extraction. The polymerase chain reaction (PCR) was performed using species-specific primers to detect *Leishmania donovani* parasites. A total of 1,662 sand flies were encountered from the entomological surveys, and the majority of them were *Phlebotomus argentipes* (*n* = 1517; 91.27%), while others were *Sergentomyia punjabiensis* (*n* = 140; 8.72%). *Leishmania donovani* parasite DNA was detected only from *P. argentipes* (2.3%; *n* = 2). The detection of *Leishmania* DNA in *P. argentipes* suggests the possible role of this species as a vector for leishmaniasis in Sri Lanka.

## 1. Introduction

Leishmaniasis is a vector-borne disease, which is caused by protozoan parasites of the genus *Leishmania* of family Trypanosomatidae and transmitted by the bite of infected adult female sand flies (Diptera: Psychodidae). It is regarded as one of the neglected tropical diseases in the world, affecting people mostly at low economic stratum in developing countries [1]. It is predicted that, globally, more than 350 million people are at risk of contracting leishmaniasis, and about two million new cases account annually [2].

Leishmaniasis has three main types of clinical presentations, namely, cutaneous leishmaniasis (CL), mucocutaneous leishmaniasis (MCL), and visceral leishmaniasis (VL). In Sri Lanka, the first indigenous case of leishmaniais was identified in 1992, which is of cutaneous origin [3].Mucocutaenous (MCL) and visceral (VL) forms have been identified in 2005 and 2007, respectively [4, 5]. At present, Sri Lanka is considered as a one of the endemic countries for cutaneous leishmaniasis in the world. The VL and MCL forms have been reported less frequently than cutaneous type so far. According to the available data, nine indigenous cases of VL and two mucosal leishmaniasis cases by the same parasite strain have been reported [6]. Currently, CL is the predominant clinical representation in the country with more than 3000 cases per year [7]. Sri Lanka has been reported as the newest focus of leishmaniasis in the Indian subcontinent, where the disease is caused by the most virulent visceralizing species, *Leishmania donovani* [8, 9]. The strain identified from Sri Lanka has only one nucleotide substitution in the glucose-6-phosphate dehydrogenase gene from *L. donovani* MON-2, the strain most commonly isolated from VL cases in India [8, 9]. The potential for visceralization in the cutaneous variant of *L. donovani* in Sri Lanka is not known until recently [9]. However, some recent studies have shown that visceralizing potential is absent in MON-37 strain identified from Sri Lanka [10]. Furthermore, studies on sylvatic and domestic reservoirs in Sri Lanka, though not entirely conclusive, indicate that this strain is more likely to be anthroponotic than zoonotic [11, 12].

Sri Lankan sand fly fauna consist of a total of 22 species from two genera: *Phlebotomus* and *Sergentomyia* [13]. The presence of *Phlebotomus* (*Euphlebotomus*) *argentipes*, the species known to be the vector for *L. donovani*, has been reported from Sri Lanka since the early 1900's [14]. However, the first confirmed indigenous CL case was reported in 1992 [3]. The confirmation of the exact vector species is a fundamental requirement in the control efforts of vector-borne diseases such as leishmaniasis [2]. The detection of the presence of parasites within the sand flies is one of the criteria to incriminate leishmaniasis vectors [2, 15]. Until recently, published information on the vectorial potential of sand flies were limited. Only two attempts in 2013 [16] and 2015 [17] have been made to confirm the presence of parasites in the field caught sand flies, which is grossly inadequate for confirmation [15].

Currently, there is no proper control program in action for leishmaniasis in Sri Lanka [18]. The detection of *Leishmania* parasites in freshly caught sand flies through traditional methods such as microscopic detection, culture, and inoculation into another animal to detect parasites within the sand fly midgut requires a high level of expertise and a relatively longer time [19, 20]. Furthermore, some of these methods are highly laborious [20, 21]. Therefore, molecular methods are more reliable and user friendly to detect *Leishmania* parasites in wild-caught sand flies [22]. Hence, the present study was conducted to detect the *Leishmania donovani* parasite DNA from wild-caught sand flies using a polymerase chain reaction (PCR) assay, which can also be used in field routine surveillance and control programs.

## 2. Methods

### 2.1. Study Area

**Figure 1:**
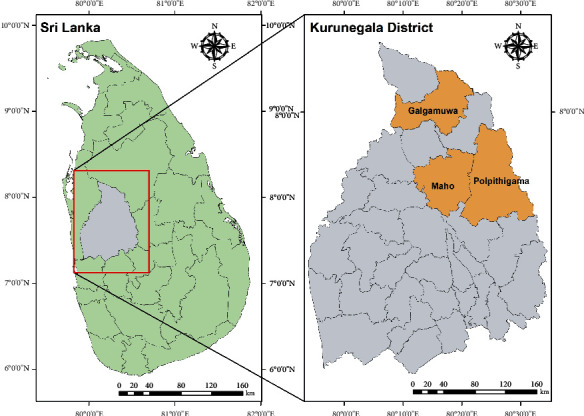
The locations of the study areas in Kurunegala District.

Kurunegala is one of the districts with the highest cutaneous leishmaniasis prevalence in Sri Lanka with a continuously increasing trend throughout the last few years [23]. At present, this district denotes the highest number of patients from the country with a prevalence rate of 0.37% [23]. It is located in the North Western Province of Sri Lanka covering a land area of 4,816 km^2^ with a human population of 1,610,299 [24]. The district receives an average of 2,095 mm rainfall annually. The average temperature and humidity is 31.7°C and 69.6%, respectively. Three Medical Officer of Health (MOH) areas in Kurunegala district, namely, Polpithigama, Maho, and Galgamuwa (Figure 1), which were considered as high-risk areas for leishmaniasis during the period of 2009–2016, were selected for the sample collections for the present study.

### 2.2. Entomological Surveillance

Entomological surveys were conducted monthly from May to October 2017 in three Medical Officer of Health (MOH) areas. Three different techniques, namely, Sticky Trap (ST), Cattle Baited Net Trap Collection (CBNT), and Hand Collection (HC) (selected indoor (bedroom, guestroom, and toilet)/outdoor (rodent burrows and wall cracks) sites) were performed to collect sand flies from three selected study locations on a monthly basis. The collections in CBNT and HC were performed using a handheld battery-operated aspirator. All traps were installed at sunset and collected during the night (8.00 pm: 12.00 midnight) and near sunrise (5.00–6.00 am).

### 2.3. Processing of Samples and Species Identification

Collected sand fly specimens were washed once in 1% detergent followed by twice in sterile distilled water. Each specimen was then dissected in a drop of fresh sterile normal saline by cutting off the head and abdominal terminalia with sterilized forceps and disposable needles. The rest of the body was stored in the sterile 1.5 ml microcentrifuge tubes and kept at −20°C freezer until use for DNA extraction. Specimens were mounted on glass slides using Hoyer's medium [25] and identified using the identification keys for species within several subgenera [25–27].

### 2.4. Extraction of Genomic DNA

The DNA was extracted using MightyPrep reagent for DNA (Takara Bio Inc, Japan) according to manufacturer's guide with some modifications as follows: a volume of 200 *μ*L from MightlyPrep reagent was added into each microcentrifuge tube and the individual sand flies were crushed using a sterile 200 *μ*L pipette tip. New pipette tips were used every time to prevent contamination of samples. The samples were subjected to hard vortex for 10 seconds followed by incubation at 95°C for 20 minutes. The samples were allowed to cool down to room temperature and a hard vortex for 10 seconds. Finally, the samples were centrifuged at 12,000 rpm for 10 minutes and stored at −20°C until used for PCR.

### 2.5. DNA Amplification

Oligonucleotide primers [F: AAATCGGCTCCGAGGCGGGAAAC; R: GGTACACTCTATCAGTAGCAC] targeting kinetoplast minicircle sequence (591 bp) of *L. donovani* as described by previous studies were used in amplification [28]. This study indicates a detection limit of 1 fg. The amplifications were conducted using 20 *μ*L of the solution containing 1 *μ*L of DNA product as the template, 2 *μ*L of CoralLoad Buffer (QIAGEN) with 15 mM MgCl_2_ and loading dye, 1.6 *μ*L of dNTP mixture with 0.2 mM from each nucleotide, 0.06 *μ*L of 0.3 *μ*M forward and reverse primers, 0.41 *μ*L of 2.5 U MightyAmp DNA Polymerase (Takara, Japan), and 15.18 *μ*L of PCR water. Amplification was performed in a thermal cycler (SimpliAmp^TM^, Applied Biosystems) programmed for an initial denaturation step of 94°C for 5 min and 40 cycles of denaturation at 94°C for 30 seconds, annealing at 50°C for 60 seconds, and extension at 72°C for 24 seconds. Nuclease-free, PCR-grade water was used as negative control and DNA isolated from *L. donovani* was used as positive controls.

### 2.6. Agarose Gel Electrophoresis

Agarose powder (Agarose S) was used for the preparation of gels with 1 X TAE. The 2% agarose gels stained with ethidium bromide (0.5 *μ*g/ml) were prepared. A volume of 4 *μ*L of the amplified PCR products was loaded with Promega 100 bp lambda marker, and gel electrophoresis was carried out at 100 V for 25 minutes to get a good separation in the amplified products. Following the gel electrophoresis, the migrated DNA was visualized and photographed under UV illumination.

### 2.7. DNA Sequencing and Confirmation of Species

The PCR amplicons were purified using QIAquick PCR Purification Kit (Qiagen), and purified products were sent to Macrogen, South Korea (Macrogen Inc., 1001, 254 Beotkkot-ro, Geumcheon-gu, Seoul, Republic of Korea) for sequencing by the Sanger method. Sequencing results were analyzed by BioEdit sequence alignment editor v7.0.9 software. The database search for homologous sequences was performed submitting kinetoplast minicircle sequences to the Basic Local Alignment Search Tool nucleotide (BLASTn) server of the National Centre for Biotechnology Information (NCBI, USA).

### 2.8. Data Analysis

The data were entered into Microsoft Excel sheets. Percentages for entomological surveillance data and molecular analysis data were calculated in Microsoft Excel. The sand fly male-to-female ratio was calculated as follows:

(1) $$\begin{array}{c} \text{male to female ratio}=\frac{\text{number of males}}{\text{number of females}}. \end{array}$$

The spatial variation of the disease prevalence was evaluated by one-way analysis of variance (ANOVA) in Minitab 17 statistical software package (Minitab Inc, Pennsylvania, USA).

## 3. Results

### 3.1. Diversity and Abundance of Sand Flies

A total of 1,662 sand flies were collected from all techniques during the study period in three MOH areas. Maho MOH area recorded the highest collection (51.81%; *n* = 861) followed by Polpithigama (40.85%; *n* = 679) and Galgamuwa (7.34%; *n* = 122). According to the morphological confirmation, two species, namely, *Phlebotomus argentipes* (*n* = 1517) and *Sergentomyia punjabiensis* (*n* = 140), were recorded. The male-to-female ratio of *P. argentipes* was 2.88 and 1.55 in *S. punjabiensis*.

The CBNT technique was contributed to the 91.28% (*n* = 1,517) of the collection followed ST (8.42%; *n* = 140) and HC (0.3%; *n* = 5). All individuals caught from CBNTs consisted only of *P. argentipes*. All sand flies collected from the STs that were placed inside houses denoted with *S. punjabiensis* only. The species composition and relative abundance are given in Table 1.

### 3.2. Molecular Detection of Parasitic DNA

**Figure 2:**
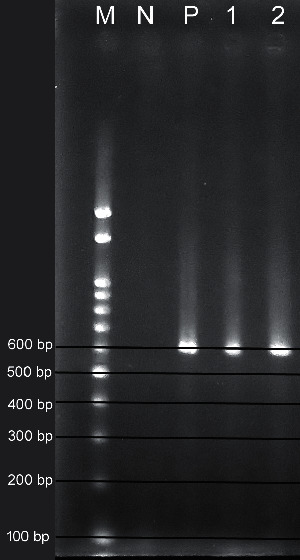
Results of the gel electrophoresis of the PCR products amplified with *Leishmania donovani* specific primers. M: 100 bp marker DNA ladder, N: negative control, P: positive control; (1) and (2) positive sand fly lysates.

The visualization of PCR products on agarose gel under UV light revealed the presence of *L. donovani* parasites only from *P. argentipes* indicating a band size at 591 bp (Figure 2).

### 3.3. Species Level Validation

**Figure 3:**
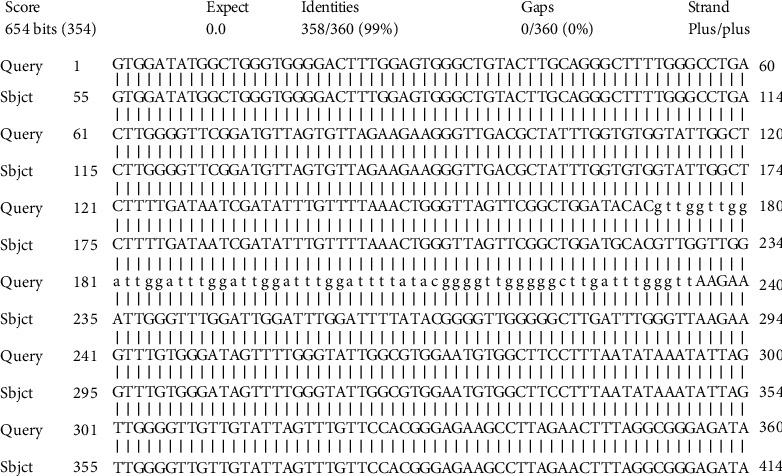
Multiple alignment of *Leishmania donovani* kinatoplast minicircle DNA sequence (query) with the sequences available in NCBI (subject).

The sequenced region of the PCR product showed 99% similarity (Figure 3) to the Sri Lankan *Leishmania* sp. Isolated from (*E* value = 0) patient samples in Sri Lanka (accession number DQ205334.1), which was later characterized as the *Leishmania donovani* zymodeme MON 37 [9].

### 3.4. Presence of *Leishmania* DNA within Sand Flies and the Leishmaniasis Incidence

According to the one-way ANOVA test, the prevalence of the disease within the last four years did not differ significantly at the 95% confidence level (*F* = 2.72; *P* > 0.05). However, the mean values for the number of cases reported during the period were highest in Polpithigama MOH area (Table 2), where the only sand flies with *L. donovani* DNA were reported during the current study.

## 4. Discussion

Investigations on the epidemiology of leishmaniasis and bioecology of sand flies are important factors for disease management and to plan control programmes [29, 30]. However, the abundance of sand flies in a leishmaniasis endemic region is merely not sufficient to characterize them as vectors responsible for transmitting leishmaniasis. One of the important criteria towards the incrimination of exact *Leishmania* vectors as per WHO guidelines is the detection of evidence for the presence of *Leishmania* DNA within sand flies [2, 31]. Dissection of the digestive tract of the sand fly is considered as the gold standard in detecting the natural infective rate. However, it is laborious and difficult in processing a large number of samples [32]. Moreover, the infection needs to be confirmed by in vitro culture of parasites or by inoculation into laboratory animals, since other nonidentified flagellates are also commonly found in the insect midgut [33]. Molecular techniques such as PCR allow for DNA detection of even a single *Leishmania* parasite [34].

In the present study, we used a PCR assay to detect *L. donovani* with species-specific primers to detect parasite DNA from field caught sand flies. These primers are designed to amplify minicircle kinetoplast DNA of the parasite. Furthermore, we compared the spatial distribution of sand flies in three MOH areas; namely, Polpithigama, Maho, and Galgamuwa in Kurunegala district. Two species, namely, *P. argentipes* and *S. punjabiensis*, were encountered from the field surveys. Of them, *P. argentipes*, the proven vector for the transmission of *L. donovani* in other countries and the main suspected vector in Sri Lanka, was predominant [16, 17]. It is interesting to note that *P. argentipes* was collected only from cattle baited net traps indicating a zoophilic nature of the species. However, the exact role of the sand flies in genus *Sergentomyia* as vectors of leishmaniasis is still uncertain and the foraging behaviour has been suggested as the main reason [35]. The species in the genus *Sergentomyia* mainly feed on cold-blooded animals, especially small reptiles such as geckoes [36, 37]. However, recent studies have confirmed that some species feed on various mammals, including humans, domesticated animals, and wild rodents [35]. Therefore, revealing of *S. punjabiensis* only from the sticky traps placed at house may indicate their endophilic resting behaviour and foraging preference to the small reptiles (geckoes), domestic animals, rodents, or may be humans. The determination of the blood meal of this species would provide the better answer as to why they prefer human habitations.

The detection limit of the primers used for the present assay was as low as 1 fg, which is equivalent of 0.1 parasites as reported by the previous studies [28]. According the published literature, the sensitivity required for the detection of parasite DNA in sand flies has been documented as 0.1–1 [38]. Thus, the primers used in this study are suitable for the detection of *L. donovani* from field-caught sand flies and results in the current study also advocate on this. The present study detected *L. donovani* DNA from 2.3% of wild-caught *P. argentipes*. Interestingly, no parasitic DNA was detected from *S. punjabiensis* suggesting that this species is unlikely to be a vector for disease transmission as indicated in the previous studies conducted in South East Asian region [30–32]. However, more extensive studies are required since the number of flies of this species collected during the current study was very low.

Molecular-based PCR assays target different conserved regions to confirm the species identification. One study conducted in Hambanthota District, Southern Province of Sri Lanka has targeted the ribosomal DNA of the *Leishmania* parasites and reported with 0.31% of *L. donovani* DNA among the wild-caught sand flies [17]. A study conducted in Brazil using ITS1 region as the target sequence has indicated 1.1% positivity [39].

The observation of a higher percentage of sand flies with *L. donovani* DNA among wild-caught sand flies from the present study may indicate a higher circulation rate of the parasite among hosts and vectors in these areas. This shows the importance of taking immediate action to conduct more extensive studies and planning effective control programs. Furthermore, once the macrophages with *Leishmania* amastigotes are ingested by sand flies along with a blood meal, the changes in the sand fly gut conditions triggers morphological transformation to promastigotes [40, 41]. The phosphoglycans on promastigote surface must bind with receptors in the midgut in order to enter the sand fly body. If the promastigote phosphoglycans are not compatible with the receptors in the sand fly midgut, the promastigotes will pass out with the fly excretions. On such occasions, the sand flies do not transmit the disease [41]. Therefore, this study does not necessarily confirm that *P. argentipes* involved in the transmission of *Leishmania* parasites. This only provides evidence for the fulfillment of a single vector incrimination criterion by *P. argentipes,* which is the circulation of *L. donovani* parasites within the sand fly population [2, 15, 42]. Although the knowledge on potential vectors was limited few years ago [18], more recent studies report that *P. argentipes* is attracted to and bites humans and potential reservoir hosts [43]. Further, this species is also known to show strong ecological associations with the leishmaniasis incidence in this area [44, 45]. Therefore, all the existing data suggest that *P. argentipes* is potentially competent vector for the transmission of *L. donovani.* The next steps should be the confirmation of luxuriant growth of the parasite within the sand fly stomdeal valve in the midgut and experimental transmission by vectors under laboratory conditions [42, 46]. The use of retrospective data to generate mathematical modeling-based proof of this species being essential for the maintenance of the parasitic transmission cycle with or without the involvement of other vectors is also a necessity. Additionally, the machine learning approaches using the results of planned control programs could be used to endorse that disease incidence significantly decreases with the biting density of the sand fly species [15].

Based on the evidence on the zoophilic nature of the main suspected vector, zooprophylaxis could be considered as a potentially effective method to reduce the infection in the human population [47–49], Furthermore, considering the zoophilic nature of *P. argentipes* as indicated by previous studies [17, 50] and the present study, it could be assumed that the disease is likely to be transmitted through an opportunistic feeding.

As described above, identification of sand flies with naturally infected *Leishmania* in more than one occasion is one of the main vector incrimination criteria [2, 15]. In this study, only 21% (95/448) of the field-caught females were randomly selected for molecular detection of the parasite. It is evidenced that 2.1% of the representative sample was positive for *Leishmania* parasite, though the sample size was low. The presence of the parasite in such a small set of sand flies could be indicative of a high rate of parasite circulation between hosts and vectors in these areas. Usually the field collections of sand flies from the surveillance activities cannot screen all collection for the presence of parasites. In terms of feasibility, cost-effectiveness, and applicability, it is good to evaluate a representative sample. Therefore, we intended to evaluate the practicality and success of such an attempt at the field setup with a representative sample to provide a user-friendly application for field surveys with evidence that could be useful for control programs.

## 5. Conclusion

The sand fly species *P. argentipes* is the most common species of sand flies found in the area, and it is the only species found to harbor *Leishmania* parasites. Therefore, it can be presumed that this species plays an important role in the transmission of leishmaniasis in Sri Lanka. Furthermore, the PCR optimized during the current study could be used as an effective method for the detection of *Leishmania* parasites residing sand fly midgut due to its user-friendliness.

## Figures and Tables

**Table 1:** Relative abundance and infection rate of sand flies.

Species	Abundance	Relative abundance (%)	Number of screened sand flies	Number of infected sand flies	Infection rate (%)	Parasite species
*P. argentipes*	1,522	91.58	87	2	2.3	*L. donovani*
*S. punjabiensis*	140	8.42	8	0	—	—
Total	1,662	100.	95	2	—	—

**Table 2:** Cutaneous leishmaniasis incidence in three selected MOH areas.

MOH area	Mean number of patients reported annually ± standrad deviation
Maho	5.50 ± 4.65
Polpithigama	15.00 ± 8.49
Galgamuwa	7.75 ± 3.86

## Data Availability

All the data generated during the study will be available from the corresponding author upon reasonable request.

## Abbreviations

ANOVA: Analysis of variance
CBNT: Cattle baited net trap
CL: Cutaneous leishmaniasis
HC: Hand collection
ITS1: Internal transcribed spacer 1
kDNA: Kinetoplast DNA
MCL: Mucocutaneous leishmaniasis
PCR: Polymerase chain reaction
ST: Sticky trap
TAE: Tris-acetate-EDTA
VL: Visceral leishmaniasis.
